# Surgical Diseases of the Ovaries and Fallopian Tubes, Including Tubal Pregnancy

**Published:** 1896-12

**Authors:** 


**Surgical Diseases of the Ovaries and Fallopian Tubes, including Tubal Pregnancy.** By J. Bland Sutton. New and enlarged Edition. Pp. xv., 428. London: Cassell and Company, Limited. 1896.

When reviewing the first edition of this book in 1893, we pointed out the great value of an intimate knowledge of pathology to the surgeon, and directed attention to the very elaborate and valuable pathological information it contained. Mr. Sutton is always collecting and examining specimens, and each edition will throw more light on the nature of diseases of the ovaries and tubes. The originality of the work which was so evident in the first edition is as marked a feature of the additions in this later one. Mr. Sutton tells us that his object in writing it has been "to enable deduction from observation to replace hypothesis and speculation," and certainly deductions from observations of the most valuable kind are abundant. Like so many other standard works, it originated in an essay which obtained the Jacksonian prize.

We notice in this new edition a development of clinical gynæcology, such as we might expect from Mr. Sutton's new appointment as Surgeon to the Chelsea Hospital for Women. The much debated question as to the effects of the removal of the ovaries on the secondary sexual characters of women is discussed on a basis of careful and extended observation, and the, possibly, more important subject of the effect of removal of the ovaries and tubes on menstruation is fully considered. The new method of treating osteomalacia by removal of the ovaries is referred to and the connection between this disease and uterine function discussed.

Not only are all forms of ovarian tumours very fully and clearly described, with the aid of a splendid series of illustrations, but the practical details of ovariotomy are given in a way which will greatly assist those who are not very experienced in the operation. Mr. Sutton's suggestion to use what he calls "the tell-tale" sponge seems to us distinctly valuable. The sponge on a holder is pushed down into the pelvis and allowed to remain there while the sutures are put in, and on withdrawal before tying the sutures, it informs us if any bleeding is going on.

The subject of tubal gestation is very fully treated, both from its pathological and clinical aspects. Mr. Sutton has collected all the English cases in which tubal pregnancy has been diagnosed before primary rupture, and removed. There are ten such. The first case was diagnosed by Herman in 1890. The author's other tables of cases of extra-uterine gestation at later periods are very valuable, but they would certainly have been much more so had he not confined himself to the records of British surgeons. The difficulties and dangers of dealing with the placenta in operations during the later months of extra-uterine pregnancy are graphically described.

The appearance of this new edition of Mr. Bland Sutton's book should be an incentive to every surgeon to re-read such a valuable and instructive work.

